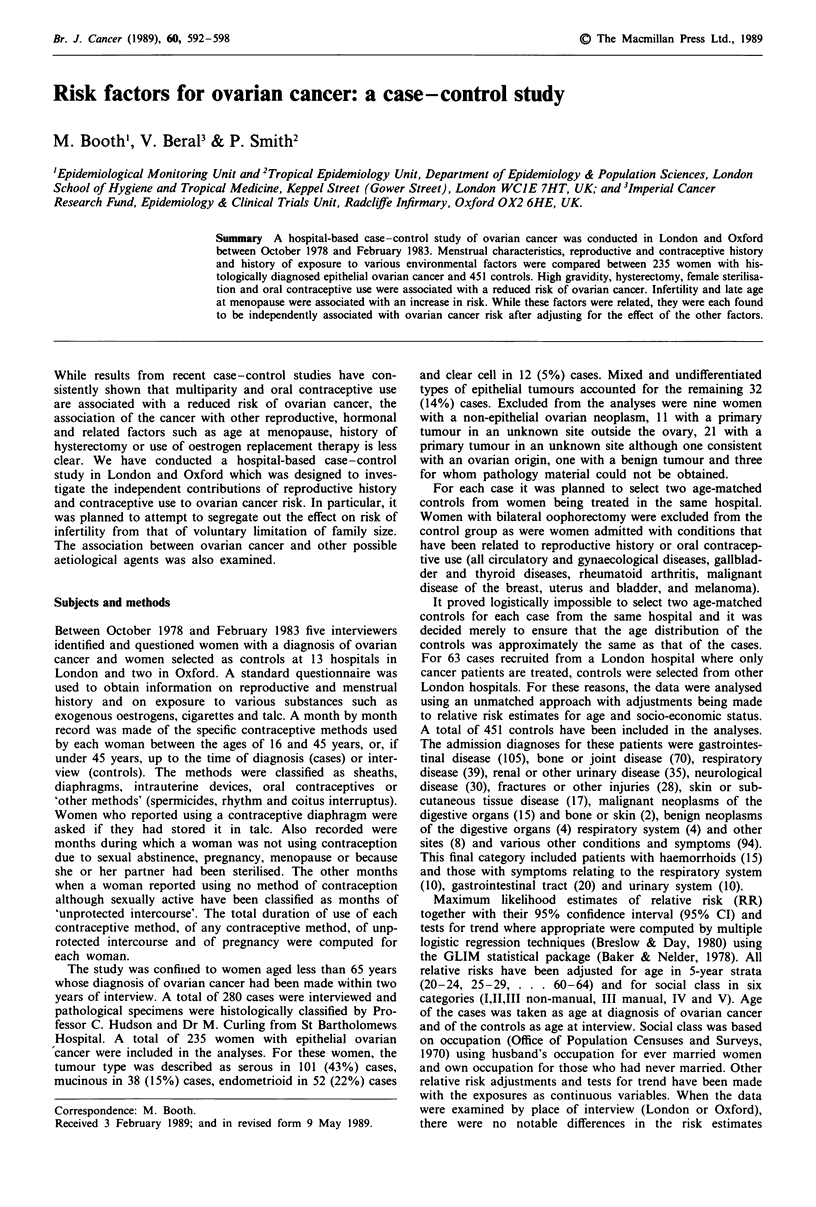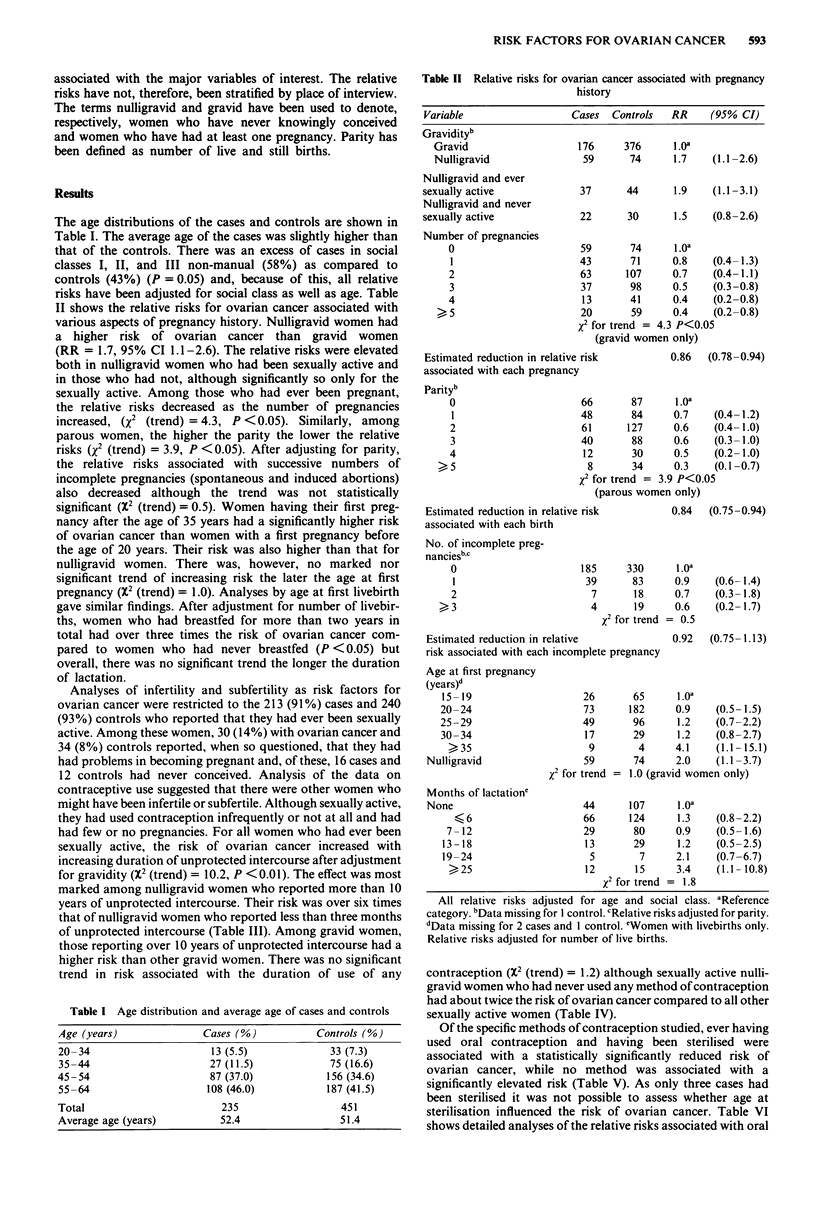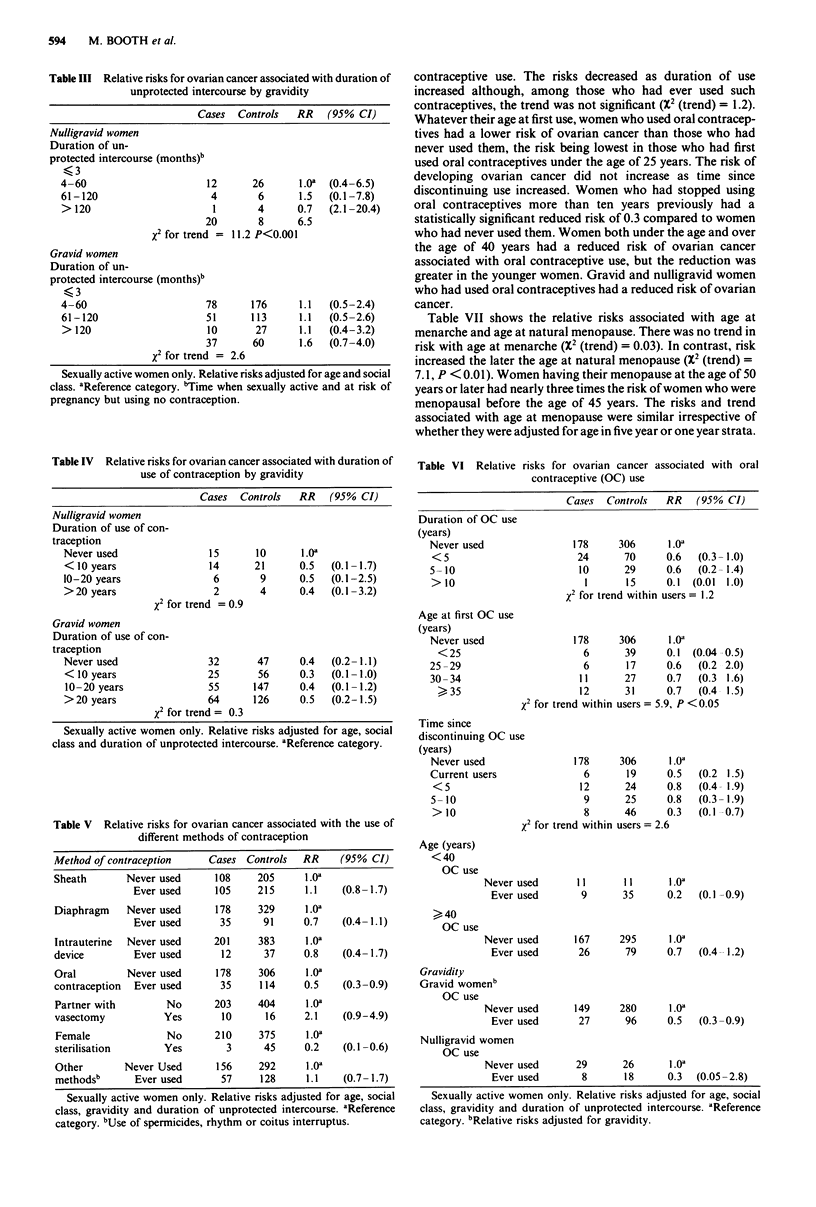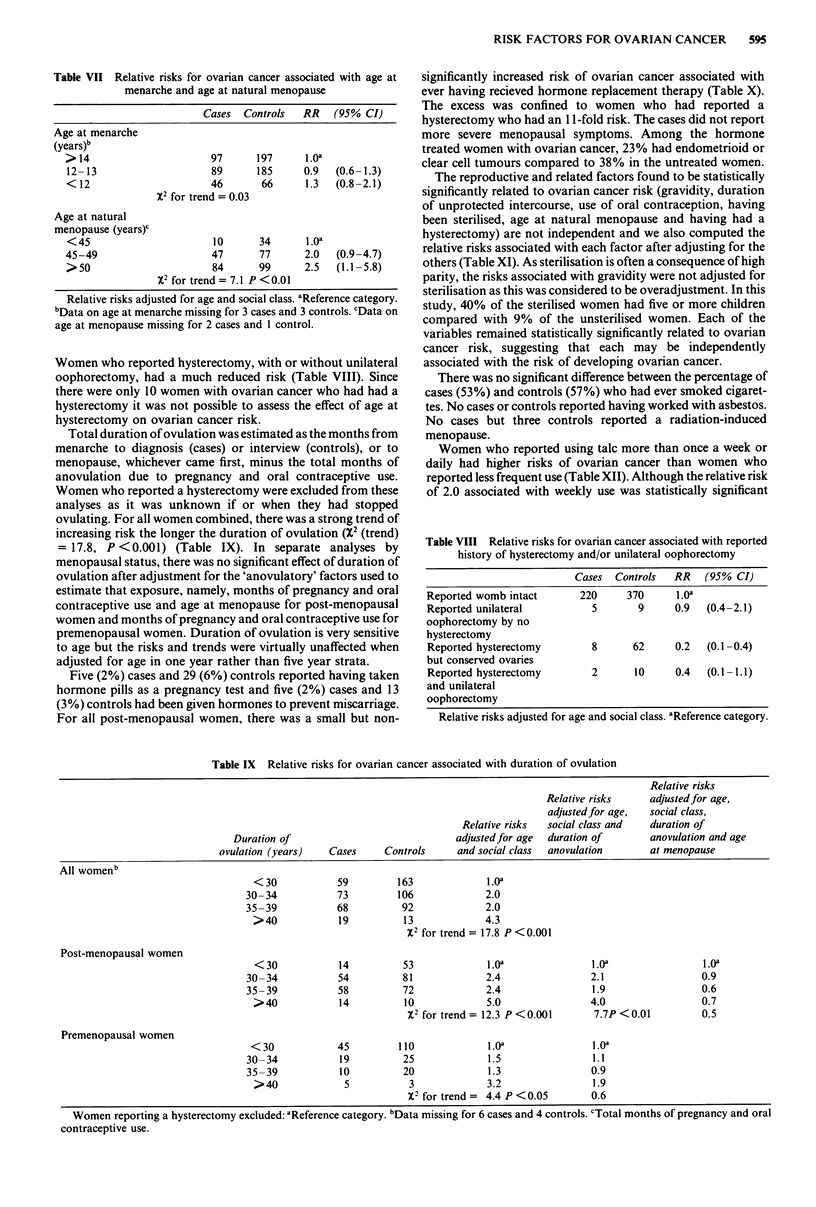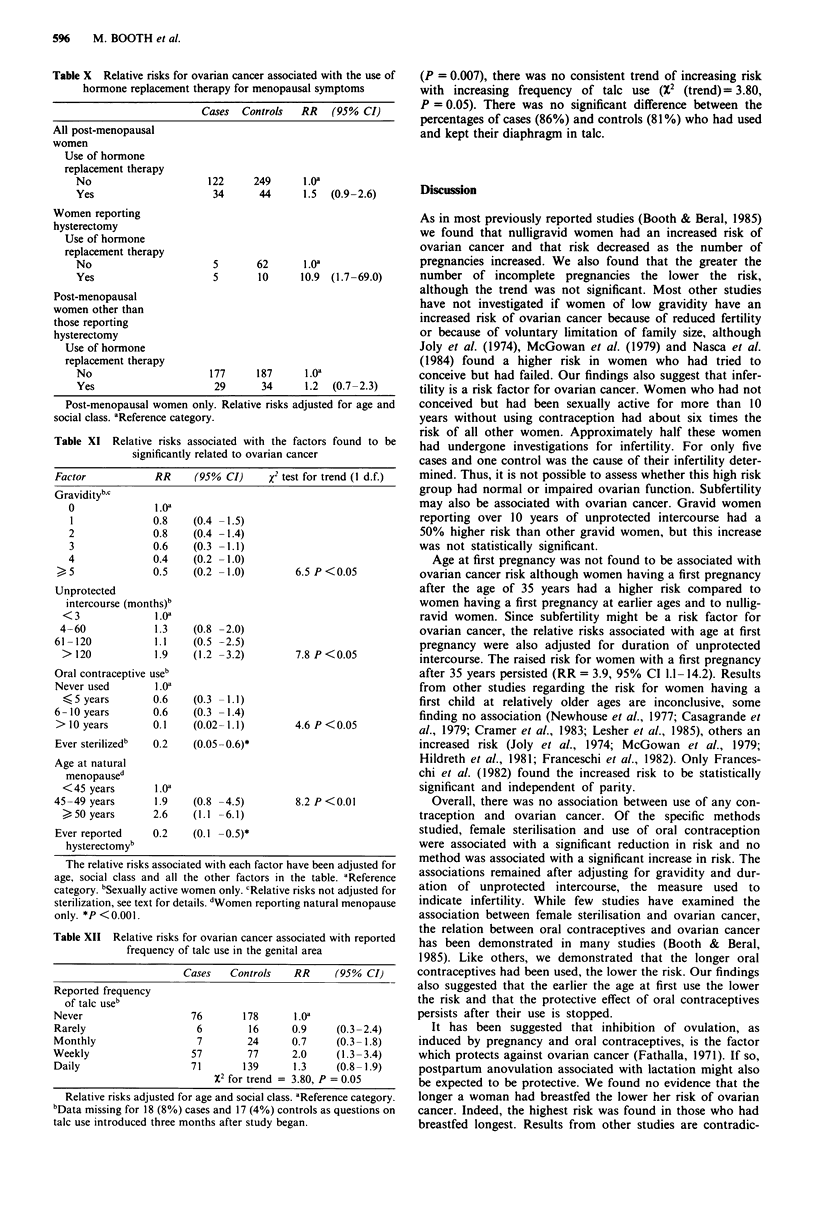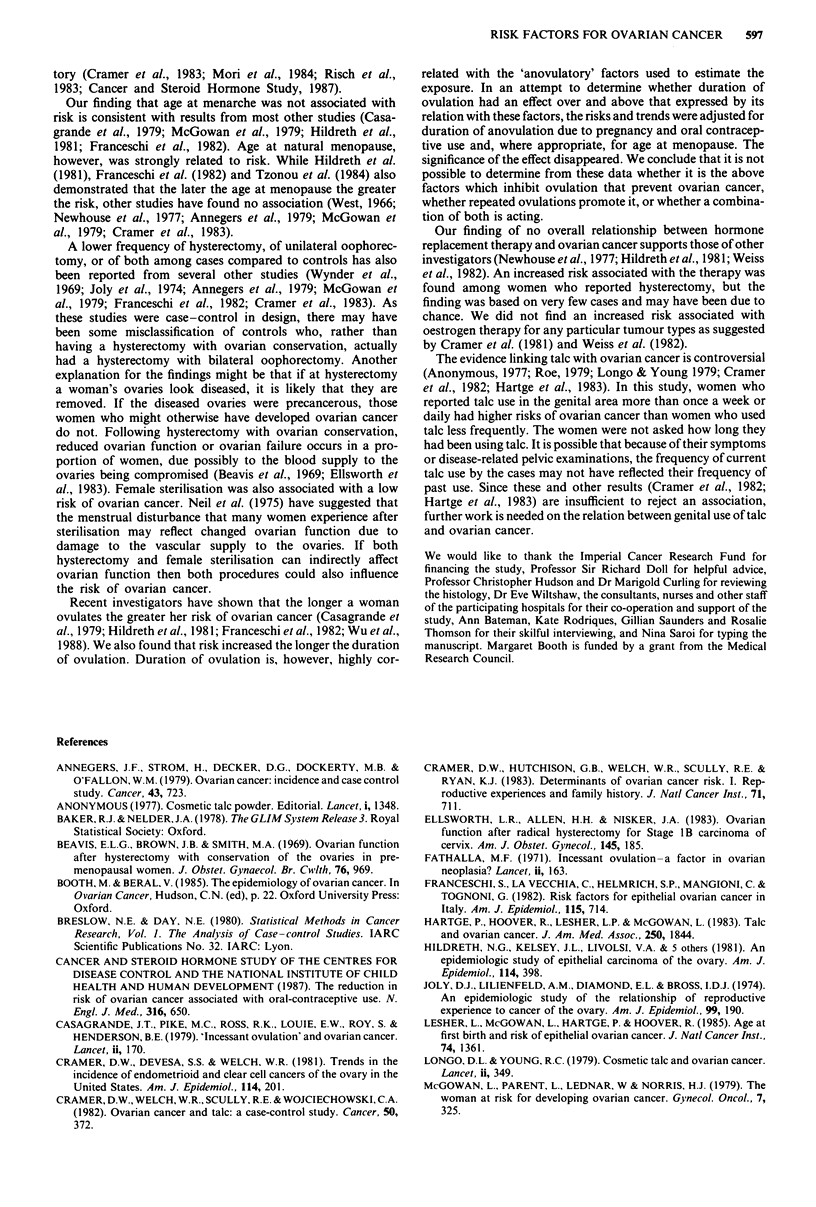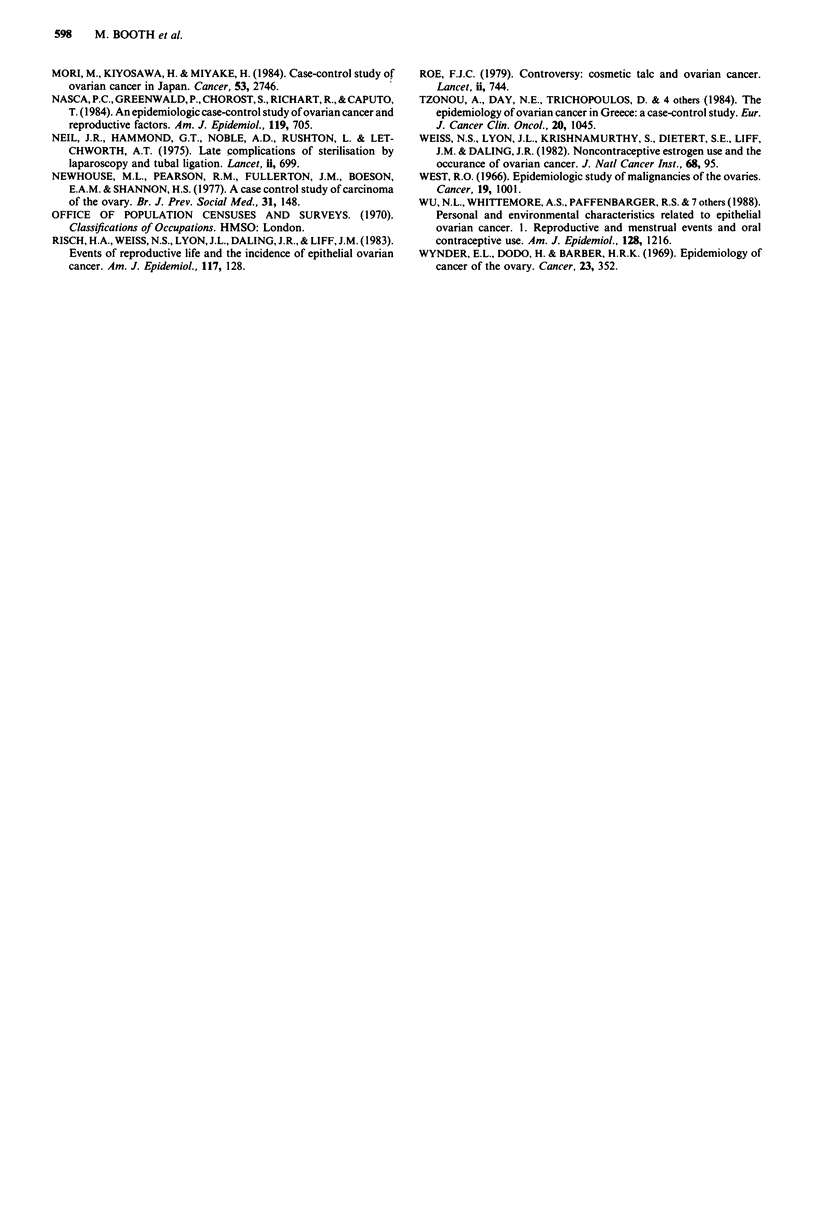# Risk factors for ovarian cancer: a case-control study.

**DOI:** 10.1038/bjc.1989.320

**Published:** 1989-10

**Authors:** M. Booth, V. Beral, P. Smith

**Affiliations:** Department of Epidemiology & Population Sciences, London School of Hygiene and Tropical Medicine, UK.

## Abstract

A hospital-based case-control study of ovarian cancer was conducted in London and Oxford between October 1978 and February 1983. Menstrual characteristics, reproductive and contraceptive history and history of exposure to various environmental factors were compared between 235 women with histologically diagnosed epithelial ovarian cancer and 451 controls. High gravidity, hysterectomy, female sterilisation and oral contraceptive use were associated with a reduced risk of ovarian cancer. Infertility and late age at menopause were associated with an increase in risk. While these factors were related, they were each found to be independently associated with ovarian cancer risk after adjusting for the effect of the other factors.


Br. J. Cancer (1989), 60, 592–598 © The Macmillan Press Ltd., 1989

### Risk factors for ovarian cancer: a case–control study

M. Booth[1], V. Beral[3] & P. Smith[2]

[1]*Epidemiological Monitoring Unit and* [2]*Tropical Epidemiology Unit, Department of Epidemiology & Population Sciences, London School of Hygiene and Tropical Medicine, Keppel Street (Gower Street), London WC1E 7HT, UK; and* [3]*Imperial Cancer Research Fund, Epidemiology & Clinical Trials Unit, Radcliffe Infirmary, Oxford OX2 6HE, UK.*

**Summary** A hospital-based case–control study of ovarian cancer was conducted in London and Oxford between October 1978 and February 1983. Menstrual characteristics, reproductive and contraceptive history and history of exposure to various environmental factors were compared between 235 women with histologically diagnosed epithelial ovarian cancer and 451 controls. High gravidity, hysterectomy, female sterilisation and oral contraceptive use were associated with a reduced risk of ovarian cancer. Infertility and late age at menopause were associated with an increase in risk. While these factors were related, they were each found to be independently associated with ovarian cancer risk after adjusting for the effect of the other factors.

While results from recent case–control studies have consistently shown that multiparity and oral contraceptive use are associated with a reduced risk of ovarian cancer, the association of the cancer with other reproductive, hormonal and related factors such as age at menopause, history of hysterectomy or use of oestrogen replacement therapy is less clear. We have conducted a hospital-based case–control study in London and Oxford which was designed to investigate the independent contributions of reproductive history and contraceptive use to ovarian cancer risk. In particular, it was planned to attempt to segregate out the effect on risk of infertility from that of voluntary limitation of family size. The association between ovarian cancer and other possible aetiological agents was also examined.

### Subjects and methods

Between October 1978 and February 1983 five interviewers identified and questioned women with a diagnosis of ovarian cancer and women selected as controls at 13 hospitals in London and two in Oxford. A standard questionnaire was used to obtain information on reproductive and menstrual history and on exposure to various substances such as exogenous oestrogens, cigarettes and talc. A month by month record was made of the specific contraceptive methods used by each woman between the ages of 16 and 45 years, or, if under 45 years, up to the time of diagnosis (cases) or interview (controls). The methods were classified as sheaths, diaphragms, intrauterine devices, oral contraceptives or 'other methods' (spermicides, rhythm and coitus interruptus). Women who reported using a contraceptive diaphragm were asked if they had stored it in talc. Also recorded were months during which a woman was not using contraception due to sexual abstinence, pregnancy, menopause or because she or her partner had been sterilised. The other months when a woman reported using no method of contraception although sexually active have been classified as months of 'unprotected intercourse'. The total duration of use of each contraceptive method, of any contraceptive method, of unprotected intercourse and of pregnancy were computed for each woman.

The study was confined to women aged less than 65 years whose diagnosis of ovarian cancer had been made within two years of interview. A total of 280 cases were interviewed and pathological specimens were histologically classified by Professor C. Hudson and Dr M. Curling from St Bartholomews Hospital. A total of 235 women with epithelial ovarian cancer were included in the analyses. For these women, the tumour type was described as serous in 101 (43%) cases, mucinous in 38 (15%) cases, endometrioid in 52 (22%) cases and clear cell in 12 (5%) cases. Mixed and undifferentiated types of epithelial tumours accounted for the remaining 32 (14%) cases. Excluded from the analyses were nine women with a non-epithelial ovarian neoplasm, 11 with a primary tumour in an unknown site outside the ovary, 21 with a primary tumour in an unknown site although one consistent with an ovarian origin, one with a benign tumour and three for whom pathology material could not be obtained.

For each case it was planned to select two age-matched controls from women being treated in the same hospital. Women with bilateral oophorectomy were excluded from the control group as were women admitted with conditions that have been related to reproductive history or oral contraceptive use (all circulatory and gynaecological diseases, gallbladder and thyroid diseases, rheumatoid arthritis, malignant disease of the breast, uterus and bladder, and melanoma).

It proved logistically impossible to select two age-matched controls for each case from the same hospital and it was decided merely to ensure that the age distribution of the controls was approximately the same as that of the cases. For 63 cases recruited from a London hospital where only cancer patients are treated, controls were selected from other London hospitals. For these reasons, the data were analysed using an unmatched approach with adjustments being made to relative risk estimates for age and socio-economic status. A total of 451 controls have been included in the analyses. The admission diagnoses for these patients were gastrointestinal disease (105), bone or joint disease (70), respiratory disease (39), renal or other urinary disease (35), neurological disease (30), fractures or other injuries (28), skin or subcutaneous tissue disease (17), malignant neoplasms of the digestive organs (15) and bone or skin (2), benign neoplasms of the digestive organs (4) respiratory system (4) and other sites (8) and various other conditions and symptoms (94). This final category included patients with haemorrhoids (15) and those with symptoms relating to the respiratory system (10), gastrointestinal tract (20) and urinary system (10).

Maximum likelihood estimates of relative risk (RR) together with their 95% confidence interval (95% CI) and tests for trend where appropriate were computed by multiple logistic regression techniques (Breslow & Day, 1980) using the GLIM statistical package (Baker & Nelder, 1978). All relative risks have been adjusted for age in 5-year strata (20–24, 25–29, . . . 60–64) and for social class in six categories (I,II,III non-manual, III manual, IV and V). Age of the cases was taken as age at diagnosis of ovarian cancer and of the controls as age at interview. Social class was based on occupation (Office of Population Censuses and Surveys, 1970) using husband's occupation for ever married women and own occupation for those who had never married. Other relative risk adjustments and tests for trend have been made with the exposures as continuous variables. When the data were examined by place of interview (London or Oxford), there were no notable differences in the risk estimates

Correspondence: M. Booth.
Received 3 February 1989; and in revised form 9 May 1989.

associated with the major variables of interest. The relative risks have not, therefore, been stratified by place of interview. The terms nulligravid and gravid have been used to denote, respectively, women who have never knowingly conceived and women who have had at least one pregnancy. Parity has been defined as number of live and still births.

### Results

The age distributions of the cases and controls are shown in Table I. The average age of the cases was slightly higher than that of the controls. There was an excess of cases in social classes I, II, and III non-manual (58%) as compared to controls (43%) ($P = 0.05$) and, because of this, all relative risks have been adjusted for social class as well as age. Table II shows the relative risks for ovarian cancer associated with various aspects of pregnancy history. Nulligravid women had a higher risk of ovarian cancer than gravid women (RR = 1.7, 95% CI 1.1–2.6). The relative risks were elevated both in nulligravid women who had been sexually active and in those who had not, although significantly so only for the sexually active. Among those who had ever been pregnant, the relative risks decreased as the number of pregnancies increased, ($\chi^2$ (trend) = 4.3, $P < 0.05$). Similarly, among parous women, the higher the parity the lower the relative risks ($\chi^2$ (trend) = 3.9, $P < 0.05$). After adjusting for parity, the relative risks associated with successive numbers of incomplete pregnancies (spontaneous and induced abortions) also decreased although the trend was not statistically significant ($\chi^2$ (trend) = 0.5). Women having their first pregnancy after the age of 35 years had a significantly higher risk of ovarian cancer than women with a first pregnancy before the age of 20 years. Their risk was also higher than that for nulligravid women. There was, however, no marked nor significant trend of increasing risk the later the age at first pregnancy ($\chi^2$ (trend) = 1.0). Analyses by age at first livebirth gave similar findings. After adjustment for number of livebirths, women who had breastfed for more than two years in total had over three times the risk of ovarian cancer compared to women who had never breastfed ($P < 0.05$) but overall, there was no significant trend the longer the duration of lactation.

Analyses of infertility and subfertility as risk factors for ovarian cancer were restricted to the 213 (91%) cases and 240 (93%) controls who reported that they had ever been sexually active. Among these women, 30 (14%) with ovarian cancer and 34 (8%) controls reported, when so questioned, that they had had problems in becoming pregnant and, of these, 16 cases and 12 controls had never conceived. Analysis of the data on contraceptive use suggested that there were other women who might have been infertile or subfertile. Although sexually active, they had used contraception infrequently or not at all and had had few or no pregnancies. For all women who had ever been sexually active, the risk of ovarian cancer increased with increasing duration of unprotected intercourse after adjustment for gravidity ($\chi^2$ (trend) = 10.2, $P < 0.01$). The effect was most marked among nulligravid women who reported more than 10 years of unprotected intercourse. Their risk was over six times that of nulligravid women who reported less than three months of unprotected intercourse (Table III). Among gravid women, those reporting over 10 years of unprotected intercourse had a higher risk than other gravid women. There was no significant trend in risk associated with the duration of use of any

**Table I** Age distribution and average age of cases and controls

| *Age (years)* | *Cases (%)* | *Controls (%)* |
|---|---|---|
| 20–34 | 13 (5.5) | 33 (7.3) |
| 35–44 | 27 (11.5) | 75 (16.6) |
| 45–54 | 87 (37.0) | 156 (34.6) |
| 55–64 | 108 (46.0) | 187 (41.5) |
| Total | 235 | 451 |
| Average age (years) | 52.4 | 51.4 |

**Table II** Relative risks for ovarian cancer associated with pregnancy history

| *Variable* | *Cases* | *Controls* | *RR* | *(95% CI)* |
|---|---|---|---|---|
| Gravidity[b] | | | | |
| Gravid | 176 | 376 | 1.0[a] | |
| Nulligravid | 59 | 74 | 1.7 | (1.1–2.6) |
| Nulligravid and ever sexually active | 37 | 44 | 1.9 | (1.1–3.1) |
| Nulligravid and never sexually active | 22 | 30 | 1.5 | (0.8–2.6) |
| Number of pregnancies | | | | |
| 0 | 59 | 74 | 1.0[a] | |
| 1 | 43 | 71 | 0.8 | (0.4–1.3) |
| 2 | 63 | 107 | 0.7 | (0.4–1.1) |
| 3 | 37 | 98 | 0.5 | (0.3–0.8) |
| 4 | 13 | 41 | 0.4 | (0.2–0.8) |
| ≥5 | 20 | 59 | 0.4 | (0.2–0.8) |
| | $\chi^2$ for trend = 4.3 $P<0.05$ (gravid women only) | | | |
| Estimated reduction in relative risk associated with each pregnancy | | | 0.86 | (0.78–0.94) |
| Parity[b] | | | | |
| 0 | 66 | 87 | 1.0[a] | |
| 1 | 48 | 84 | 0.7 | (0.4–1.2) |
| 2 | 61 | 127 | 0.6 | (0.4–1.0) |
| 3 | 40 | 88 | 0.6 | (0.3–1.0) |
| 4 | 12 | 30 | 0.5 | (0.2–1.0) |
| ≥5 | 8 | 34 | 0.3 | (0.1–0.7) |
| | $\chi^2$ for trend = 3.9 $P<0.05$ (parous women only) | | | |
| Estimated reduction in relative risk associated with each birth | | | 0.84 | (0.75–0.94) |
| No. of incomplete pregnancies[b,c] | | | | |
| 0 | 185 | 330 | 1.0[a] | |
| 1 | 39 | 83 | 0.9 | (0.6–1.4) |
| 2 | 7 | 18 | 0.7 | (0.3–1.8) |
| ≥3 | 4 | 19 | 0.6 | (0.2–1.7) |
| | $\chi^2$ for trend = 0.5 | | | |
| Estimated reduction in relative risk associated with each incomplete pregnancy | | | 0.92 | (0.75–1.13) |
| Age at first pregnancy (years)[d] | | | | |
| 15–19 | 26 | 65 | 1.0[a] | |
| 20–24 | 73 | 182 | 0.9 | (0.5–1.5) |
| 25–29 | 49 | 96 | 1.2 | (0.7–2.2) |
| 30–34 | 17 | 29 | 1.2 | (0.8–2.7) |
| ≥35 | 9 | 4 | 4.1 | (1.1–15.1) |
| Nulligravid | 59 | 74 | 2.0 | (1.1–3.7) |
| | $\chi^2$ for trend = 1.0 (gravid women only) | | | |
| Months of lactation[e] | | | | |
| None | 44 | 107 | 1.0[a] | |
| ≤6 | 66 | 124 | 1.3 | (0.8–2.2) |
| 7–12 | 29 | 80 | 0.9 | (0.5–1.6) |
| 13–18 | 13 | 29 | 1.2 | (0.5–2.5) |
| 19–24 | 5 | 7 | 2.1 | (0.7–6.7) |
| ≥25 | 12 | 15 | 3.4 | (1.1–10.8) |
| | $\chi^2$ for trend = 1.8 | | | |

All relative risks adjusted for age and social class. [a]Reference category. [b]Data missing for 1 control. [c]Relative risks adjusted for parity. [d]Data missing for 2 cases and 1 control. [e]Women with livebirths only. Relative risks adjusted for number of live births.

contraception ($\chi^2$ (trend) = 1.2) although sexually active nulligravid women who had never used any method of contraception had about twice the risk of ovarian cancer compared to all other sexually active women (Table IV).

Of the specific methods of contraception studied, ever having used oral contraception and having been sterilised were associated with a statistically significantly reduced risk of ovarian cancer, while no method was associated with a significantly elevated risk (Table V). As only three cases had been sterilised it was not possible to assess whether age at sterilisation influenced the risk of ovarian cancer. Table VI shows detailed analyses of the relative risks associated with oral contraceptive use. The risks decreased as duration of use increased although, among those who had ever used such contraceptives, the trend was not significant ($\chi^2$ (trend) = 1.2). Whatever their age at first use, women who used oral contraceptives had a lower risk of ovarian cancer than those who had never used them, the risk being lowest in those who had first used oral contraceptives under the age of 25 years. The risk of developing ovarian cancer did not increase as time since discontinuing use increased. Women who had stopped using oral contraceptives more than ten years previously had a statistically significant reduced risk of 0.3 compared to women who had never used them. Women both under the age and over the age of 40 years had a reduced risk of ovarian cancer associated with oral contraceptive use, but the reduction was greater in the younger women. Gravid and nulligravid women who had used oral contraceptives had a reduced risk of ovarian cancer.

Table VII shows the relative risks associated with age at menarche and age at natural menopause. There was no trend in risk with age at menarche ($\chi^2$ (trend) = 0.03). In contrast, risk increased the later the age at natural menopause ($\chi^2$ (trend) = 7.1, $P<0.01$). Women having their menopause at the age of 50 years or later had nearly three times the risk of women who were menopausal before the age of 45 years. The risks and trend associated with age at menopause were similar irrespective of whether they were adjusted for age in five year or one year strata.

**Table III** Relative risks for ovarian cancer associated with duration of unprotected intercourse by gravidity

| | Cases | Controls | RR | (95% CI) |
|---|---|---|---|---|
| *Nulligravid women* | | | | |
| Duration of un-protected intercourse (months)[b] | | | | |
| ≤3 | | | | |
| 4–60 | 12 | 26 | 1.0[a] | (0.4–6.5) |
| 61–120 | 4 | 6 | 1.5 | (0.1–7.8) |
| >120 | 1 | 4 | 0.7 | (2.1–20.4) |
| | 20 | 8 | 6.5 | |
| $\chi^2$ for trend = 11.2 $P<0.001$ | | | | |
| *Gravid women* | | | | |
| Duration of un-protected intercourse (months)[b] | | | | |
| ≤3 | | | | |
| 4–60 | 78 | 176 | 1.1 | (0.5–2.4) |
| 61–120 | 51 | 113 | 1.1 | (0.5–2.6) |
| >120 | 10 | 27 | 1.1 | (0.4–3.2) |
| | 37 | 60 | 1.6 | (0.7–4.0) |
| $\chi^2$ for trend = 2.6 | | | | |

Sexually active women only. Relative risks adjusted for age and social class. [a]Reference category. [b]Time when sexually active and at risk of pregnancy but using no contraception.

**Table IV** Relative risks for ovarian cancer associated with duration of use of contraception by gravidity

| | Cases | Controls | RR | (95% CI) |
|---|---|---|---|---|
| *Nulligravid women* | | | | |
| Duration of use of con-traception | | | | |
| Never used | 15 | 10 | 1.0[a] | |
| <10 years | 14 | 21 | 0.5 | (0.1–1.7) |
| 10–20 years | 6 | 9 | 0.5 | (0.1–2.5) |
| >20 years | 2 | 4 | 0.4 | (0.1–3.2) |
| $\chi^2$ for trend = 0.9 | | | | |
| *Gravid women* | | | | |
| Duration of use of con-traception | | | | |
| Never used | 32 | 47 | 0.4 | (0.2–1.1) |
| <10 years | 25 | 56 | 0.3 | (0.1–1.0) |
| 10–20 years | 55 | 147 | 0.4 | (0.1–1.2) |
| >20 years | 64 | 126 | 0.5 | (0.2–1.5) |
| $\chi^2$ for trend = 0.3 | | | | |

Sexually active women only. Relative risks adjusted for age, social class and duration of unprotected intercourse. [a]Reference category.

**Table V** Relative risks for ovarian cancer associated with the use of different methods of contraception

| Method of contraception | | Cases | Controls | RR | (95% CI) |
|---|---|---|---|---|---|
| Sheath | Never used | 108 | 205 | 1.0[a] | |
| | Ever used | 105 | 215 | 1.1 | (0.8–1.7) |
| Diaphragm | Never used | 178 | 329 | 1.0[a] | |
| | Ever used | 35 | 91 | 0.7 | (0.4–1.1) |
| Intrauterine | Never used | 201 | 383 | 1.0[a] | |
| device | Ever used | 12 | 37 | 0.8 | (0.4–1.7) |
| Oral | Never used | 178 | 306 | 1.0[a] | |
| contraception | Ever used | 35 | 114 | 0.5 | (0.3–0.9) |
| Partner with | No | 203 | 404 | 1.0[a] | |
| vasectomy | Yes | 10 | 16 | 2.1 | (0.9–4.9) |
| Female | No | 210 | 375 | 1.0[a] | |
| sterilisation | Yes | 3 | 45 | 0.2 | (0.1–0.6) |
| Other | Never Used | 156 | 292 | 1.0[a] | |
| methods[b] | Ever used | 57 | 128 | 1.1 | (0.7–1.7) |

Sexually active women only. Relative risks adjusted for age, social class, gravidity and duration of unprotected intercourse. [a]Reference category. [b]Use of spermicides, rhythm or coitus interruptus.

**Table VI** Relative risks for ovarian cancer associated with oral contraceptive (OC) use

| | Cases | Controls | RR | (95% CI) |
|---|---|---|---|---|
| Duration of OC use (years) | | | | |
| Never used | 178 | 306 | 1.0[a] | |
| <5 | 24 | 70 | 0.6 | (0.3–1.0) |
| 5–10 | 10 | 29 | 0.6 | (0.2–1.4) |
| >10 | 1 | 15 | 0.1 | (0.01–1.0) |
| $\chi^2$ for trend within users = 1.2 | | | | |
| Age at first OC use (years) | | | | |
| Never used | 178 | 306 | 1.0[a] | |
| <25 | 6 | 39 | 0.1 | (0.04–0.5) |
| 25–29 | 6 | 17 | 0.6 | (0.2–2.0) |
| 30–34 | 11 | 27 | 0.7 | (0.3–1.6) |
| ≥35 | 12 | 31 | 0.7 | (0.4–1.5) |
| $\chi^2$ for trend within users = 5.9, $P<0.05$ | | | | |
| Time since discontinuing OC use (years) | | | | |
| Never used | 178 | 306 | 1.0[a] | |
| Current users | 6 | 19 | 0.5 | (0.2–1.5) |
| <5 | 12 | 24 | 0.8 | (0.4–1.9) |
| 5–10 | 9 | 25 | 0.8 | (0.3–1.9) |
| >10 | 8 | 46 | 0.3 | (0.1–0.7) |
| $\chi^2$ for trend within users = 2.6 | | | | |
| Age (years) | | | | |
| <40 | | | | |
| OC use | | | | |
| Never used | 11 | 11 | 1.0[a] | |
| Ever used | 9 | 35 | 0.2 | (0.1–0.9) |
| ≥40 | | | | |
| OC use | | | | |
| Never used | 167 | 295 | 1.0[a] | |
| Ever used | 26 | 79 | 0.7 | (0.4–1.2) |
| *Gravidity* | | | | |
| Gravid women[b] | | | | |
| OC use | | | | |
| Never used | 149 | 280 | 1.0[a] | |
| Ever used | 27 | 96 | 0.5 | (0.3–0.9) |
| Nulligravid women | | | | |
| OC use | | | | |
| Never used | 29 | 26 | 1.0[a] | |
| Ever used | 8 | 18 | 0.3 | (0.05–2.8) |

Sexually active women only. Relative risks adjusted for age, social class, gravidity and duration of unprotected intercourse. [a]Reference category. [b]Relative risks adjusted for gravidity.

**Table VII** Relative risks for ovarian cancer associated with age at menarche and age at natural menopause

| | *Cases* | *Controls* | *RR* | *(95% CI)* |
|---|---|---|---|---|
| Age at menarche (years)[b] | | | | |
| >14 | 97 | 197 | 1.0[a] | |
| 12–13 | 89 | 185 | 0.9 | (0.6–1.3) |
| <12 | 46 | 66 | 1.3 | (0.8–2.1) |
| $\chi^2$ for trend = 0.03 | | | | |
| Age at natural menopause (years)[c] | | | | |
| <45 | 10 | 34 | 1.0[a] | |
| 45–49 | 47 | 77 | 2.0 | (0.9–4.7) |
| >50 | 84 | 99 | 2.5 | (1.1–5.8) |
| $\chi^2$ for trend = 7.1 $P<0.01$ | | | | |

Relative risks adjusted for age and social class. [a]Reference category. [b]Data on age at menarche missing for 3 cases and 3 controls. [c]Data on age at menopause missing for 2 cases and 1 control.

Women who reported hysterectomy, with or without unilateral oophorectomy, had a much reduced risk (Table VIII). Since there were only 10 women with ovarian cancer who had had a hysterectomy it was not possible to assess the effect of age at hysterectomy on ovarian cancer risk.

Total duration of ovulation was estimated as the months from menarche to diagnosis (cases) or interview (controls), or to menopause, whichever came first, minus the total months of anovulation due to pregnancy and oral contraceptive use. Women who reported a hysterectomy were excluded from these analyses as it was unknown if or when they had stopped ovulating. For all women combined, there was a strong trend of increasing risk the longer the duration of ovulation ($\chi^2$ (trend) = 17.8, $P<0.001$) (Table IX). In separate analyses by menopausal status, there was no significant effect of duration of ovulation after adjustment for the 'anovulatory' factors used to estimate that exposure, namely, months of pregnancy and oral contraceptive use and age at menopause for post-menopausal women and months of pregnancy and oral contraceptive use for premenopausal women. Duration of ovulation is very sensitive to age but the risks and trends were virtually unaffected when adjusted for age in one year rather than five year strata.

Five (2%) cases and 29 (6%) controls reported having taken hormone pills as a pregnancy test and five (2%) cases and 13 (3%) controls had been given hormones to prevent miscarriage. For all post-menopausal women, there was a small but non-significantly increased risk of ovarian cancer associated with ever having recieved hormone replacement therapy (Table X). The excess was confined to women who had reported a hysterectomy who had an 11-fold risk. The cases did not report more severe menopausal symptoms. Among the hormone treated women with ovarian cancer, 23% had endometrioid or clear cell tumours compared to 38% in the untreated women.

The reproductive and related factors found to be statistically significantly related to ovarian cancer risk (gravidity, duration of unprotected intercourse, use of oral contraception, having been sterilised, age at natural menopause and having had a hysterectomy) are not independent and we also computed the relative risks associated with each factor after adjusting for the others (Table XI). As sterilisation is often a consequence of high parity, the risks associated with gravidity were not adjusted for sterilisation as this was considered to be overadjustment. In this study, 40% of the sterilised women had five or more children compared with 9% of the unsterilised women. Each of the variables remained statistically significantly related to ovarian cancer risk, suggesting that each may be independently associated with the risk of developing ovarian cancer.

There was no significant difference between the percentage of cases (53%) and controls (57%) who had ever smoked cigarettes. No cases or controls reported having worked with asbestos. No cases but three controls reported a radiation-induced menopause.

Women who reported using talc more than once a week or daily had higher risks of ovarian cancer than women who reported less frequent use (Table XII). Although the relative risk of 2.0 associated with weekly use was statistically significant

**Table VIII** Relative risks for ovarian cancer associated with reported history of hysterectomy and/or unilateral oophorectomy

| | *Cases* | *Controls* | *RR* | *(95% CI)* |
|---|---|---|---|---|
| Reported womb intact | 220 | 370 | 1.0[a] | |
| Reported unilateral oophorectomy by no hysterectomy | 5 | 9 | 0.9 | (0.4–2.1) |
| Reported hysterectomy but conserved ovaries | 8 | 62 | 0.2 | (0.1–0.4) |
| Reported hysterectomy and unilateral oophorectomy | 2 | 10 | 0.4 | (0.1–1.1) |

Relative risks adjusted for age and social class. [a]Reference category.

**Table IX** Relative risks for ovarian cancer associated with duration of ovulation

| | *Duration of ovulation (years)* | *Cases* | *Controls* | *Relative risks adjusted for age and social class* | *Relative risks adjusted for age, social class and duration of anovulation* | *Relative risks adjusted for age, social class, duration of anovulation and age at menopause* |
|---|---|---|---|---|---|---|
| All women[b] | | | | | | |
| | <30 | 59 | 163 | 1.0[a] | | |
| | 30–34 | 73 | 106 | 2.0 | | |
| | 35–39 | 68 | 92 | 2.0 | | |
| | >40 | 19 | 13 | 4.3 | | |
| | | | $\chi^2$ for trend = | 17.8 $P<0.001$ | | |
| Post-menopausal women | | | | | | |
| | <30 | 14 | 53 | 1.0[a] | 1.0[a] | 1.0[a] |
| | 30–34 | 54 | 81 | 2.4 | 2.1 | 0.9 |
| | 35–39 | 58 | 72 | 2.4 | 1.9 | 0.6 |
| | >40 | 14 | 10 | 5.0 | 4.0 | 0.7 |
| | | | $\chi^2$ for trend = | 12.3 $P<0.001$ | 7.7$P<0.01$ | 0.5 |
| Premenopausal women | | | | | | |
| | <30 | 45 | 110 | 1.0[a] | 1.0[a] | |
| | 30–34 | 19 | 25 | 1.5 | 1.1 | |
| | 35–39 | 10 | 20 | 1.3 | 0.9 | |
| | >40 | 5 | 3 | 3.2 | 1.9 | |
| | | | $\chi^2$ for trend = | 4.4 $P<0.05$ | 0.6 | |

Women reporting a hysterectomy excluded: [a]Reference category. [b]Data missing for 6 cases and 4 controls. [c]Total months of pregnancy and oral contraceptive use.

Table X Relative risks for ovarian cancer associated with the use of hormone replacement therapy for menopausal symptoms

| | Cases | Controls | RR | (95% CI) |
|---|---|---|---|---|
| All post-menopausal women | | | | |
| Use of hormone replacement therapy | | | | |
| No | 122 | 249 | 1.0[a] | |
| Yes | 34 | 44 | 1.5 | (0.9-2.6) |
| Women reporting hysterectomy | | | | |
| Use of hormone replacement therapy | | | | |
| No | 5 | 62 | 1.0[a] | |
| Yes | 5 | 10 | 10.9 | (1.7-69.0) |
| Post-menopausal women other than those reporting hysterectomy | | | | |
| Use of hormone replacement therapy | | | | |
| No | 177 | 187 | 1.0[a] | |
| Yes | 29 | 34 | 1.2 | (0.7-2.3) |

Post-menopausal women only. Relative risks adjusted for age and social class. [a]Reference category.

Table XI Relative risks associated with the factors found to be significantly related to ovarian cancer

| Factor | RR | (95% CI) | $\chi^2$ test for trend (1 d.f.) |
|---|---|---|---|
| Gravidity[b,c] | | | |
| 0 | 1.0[a] | | |
| 1 | 0.8 | (0.4 -1.5) | |
| 2 | 0.8 | (0.4 -1.4) | |
| 3 | 0.6 | (0.3 -1.1) | |
| 4 | 0.4 | (0.2 -1.0) | |
| ≥5 | 0.5 | (0.2 -1.0) | 6.5 $P<0.05$ |
| Unprotected intercourse (months)[b] | | | |
| <3 | 1.0[a] | | |
| 4-60 | 1.3 | (0.8 -2.0) | |
| 61-120 | 1.1 | (0.5 -2.5) | |
| >120 | 1.9 | (1.2 -3.2) | 7.8 $P<0.05$ |
| Oral contraceptive use[b] | | | |
| Never used | 1.0[a] | | |
| ≤5 years | 0.6 | (0.3 -1.1) | |
| 6-10 years | 0.6 | (0.3 -1.4) | |
| >10 years | 0.1 | (0.02-1.1) | 4.6 $P<0.05$ |
| Ever sterilized[b] | 0.2 | (0.05-0.6)* | |
| Age at natural menopause[d] | | | |
| <45 years | 1.0[a] | | |
| 45-49 years | 1.9 | (0.8 -4.5) | 8.2 $P<0.01$ |
| ≥50 years | 2.6 | (1.1 -6.1) | |
| Ever reported hysterectomy[b] | 0.2 | (0.1 -0.5)* | |

The relative risks associated with each factor have been adjusted for age, social class and all the other factors in the table. [a]Reference category. [b]Sexually active women only. [c]Relative risks not adjusted for sterilization, see text for details. [d]Women reporting natural menopause only. *$P<0.001$.

Table XII Relative risks for ovarian cancer associated with reported frequency of talc use in the genital area

| | Cases | Controls | RR | (95% CI) |
|---|---|---|---|---|
| Reported frequency of talc use[b] | | | | |
| Never | 76 | 178 | 1.0[a] | |
| Rarely | 6 | 16 | 0.9 | (0.3-2.4) |
| Monthly | 7 | 24 | 0.7 | (0.3-1.8) |
| Weekly | 57 | 77 | 2.0 | (1.3-3.4) |
| Daily | 71 | 139 | 1.3 | (0.8-1.9) |
| | | $\chi^2$ for trend = 3.80, $P=0.05$ | | |

Relative risks adjusted for age and social class. [a]Reference category. [b]Data missing for 18 (8%) cases and 17 (4%) controls as questions on talc use introduced three months after study began.

($P=0.007$), there was no consistent trend of increasing risk with increasing frequency of talc use ($\chi^2$ (trend) = 3.80, $P=0.05$). There was no significant difference between the percentages of cases (86%) and controls (81%) who had used and kept their diaphragm in talc.

### Discussion

As in most previously reported studies (Booth & Beral, 1985) we found that nulligravid women had an increased risk of ovarian cancer and that risk decreased as the number of pregnancies increased. We also found that the greater the number of incomplete pregnancies the lower the risk, although the trend was not significant. Most other studies have not investigated if women of low gravidity have an increased risk of ovarian cancer because of reduced fertility or because of voluntary limitation of family size, although Joly *et al.* (1974), McGowan *et al.* (1979) and Nasca *et al.* (1984) found a higher risk in women who had tried to conceive but had failed. Our findings also suggest that infertility is a risk factor for ovarian cancer. Women who had not conceived but had been sexually active for more than 10 years without using contraception had about six times the risk of all other women. Approximately half these women had undergone investigations for infertility. For only five cases and one control was the cause of their infertility determined. Thus, it is not possible to assess whether this high risk group had normal or impaired ovarian function. Subfertility may also be associated with ovarian cancer. Gravid women reporting over 10 years of unprotected intercourse had a 50% higher risk than other gravid women, but this increase was not statistically significant.

Age at first pregnancy was not found to be associated with ovarian cancer risk although women having a first pregnancy after the age of 35 years had a higher risk compared to women having a first pregnancy at earlier ages and to nulligravid women. Since subfertility might be a risk factor for ovarian cancer, the relative risks associated with age at first pregnancy were also adjusted for duration of unprotected intercourse. The raised risk for women with a first pregnancy after 35 years persisted (RR = 3.9, 95% CI 1.1-14.2). Results from other studies regarding the risk for women having a first child at relatively older ages are inconclusive, some finding no association (Newhouse *et al.*, 1977; Casagrande *et al.*, 1979; Cramer *et al.*, 1983; Lesher *et al.*, 1985), others an increased risk (Joly *et al.*, 1974; McGowan *et al.*, 1979; Hildreth *et al.*, 1981; Franceschi *et al.*, 1982). Only Franceschi *et al.* (1982) found the increased risk to be statistically significant and independent of parity.

Overall, there was no association between use of any contraception and ovarian cancer. Of the specific methods studied, female sterilisation and use of oral contraception were associated with a significant reduction in risk and no method was associated with a significant increase in risk. The associations remained after adjusting for gravidity and duration of unprotected intercourse, the measure used to indicate infertility. While few studies have examined the association between female sterilisation and ovarian cancer, the relation between oral contraceptives and ovarian cancer has been demonstrated in many studies (Booth & Beral, 1985). Like others, we demonstrated that the longer oral contraceptives had been used, the lower the risk. Our findings also suggested that the earlier the age at first use the lower the risk and that the protective effect of oral contraceptives persists after their use is stopped.

It has been suggested that inhibition of ovulation, as induced by pregnancy and oral contraceptives, is the factor which protects against ovarian cancer (Fathalla, 1971). If so, postpartum anovulation associated with lactation might also be expected to be protective. We found no evidence that the longer a woman had breastfed the lower her risk of ovarian cancer. Indeed, the highest risk was found in those who had breastfed longest. Results from other studies are contradictory (Cramer *et al.*, 1983; Mori *et al.*, 1984; Risch *et al.*, 1983; Cancer and Steroid Hormone Study, 1987).

Our finding that age at menarche was not associated with risk is consistent with results from most other studies (Casagrande *et al.*, 1979; McGowan *et al.*, 1979; Hildreth *et al.*, 1981; Franceschi *et al.*, 1982). Age at natural menopause, however, was strongly related to risk. While Hildreth *et al.* (1981), Franceschi *et al.* (1982) and Tzonou *et al.* (1984) also demonstrated that the later the age at menopause the greater the risk, other studies have found no association (West, 1966; Newhouse *et al.*, 1977; Annegers *et al.*, 1979; McGowan *et al.*, 1979; Cramer *et al.*, 1983).

A lower frequency of hysterectomy, of unilateral oophorectomy, or of both among cases compared to controls has also been reported from several other studies (Wynder *et al.*, 1969; Joly *et al.*, 1974; Annegers *et al.*, 1979; McGowan *et al.*, 1979; Franceschi *et al.*, 1982; Cramer *et al.*, 1983). As these studies were case–control in design, there may have been some misclassification of controls who, rather than having a hysterectomy with ovarian conservation, actually had a hysterectomy with bilateral oophorectomy. Another explanation for the findings might be that if at hysterectomy a woman's ovaries look diseased, it is likely that they are removed. If the diseased ovaries were precancerous, those women who might otherwise have developed ovarian cancer do not. Following hysterectomy with ovarian conservation, reduced ovarian function or ovarian failure occurs in a proportion of women, due possibly to the blood supply to the ovaries being compromised (Beavis *et al.*, 1969; Ellsworth *et al.*, 1983). Female sterilisation was also associated with a low risk of ovarian cancer. Neil *et al.* (1975) have suggested that the menstrual disturbance that many women experience after sterilisation may reflect changed ovarian function due to damage to the vascular supply to the ovaries. If both hysterectomy and female sterilisation can indirectly affect ovarian function then both procedures could also influence the risk of ovarian cancer.

Recent investigators have shown that the longer a woman ovulates the greater her risk of ovarian cancer (Casagrande *et al.*, 1979; Hildreth *et al.*, 1981; Franceschi *et al.*, 1982; Wu *et al.*, 1988). We also found that risk increased the longer the duration of ovulation. Duration of ovulation is, however, highly correlated with the 'anovulatory' factors used to estimate the exposure. In an attempt to determine whether duration of ovulation had an effect over and above that expressed by its relation with these factors, the risks and trends were adjusted for duration of anovulation due to pregnancy and oral contraceptive use and, where appropriate, for age at menopause. The significance of the effect disappeared. We conclude that it is not possible to determine from these data whether it is the above factors which inhibit ovulation that prevent ovarian cancer, whether repeated ovulations promote it, or whether a combination of both is acting.

Our finding of no overall relationship between hormone replacement therapy and ovarian cancer supports those of other investigators (Newhouse *et al.*, 1977; Hildreth *et al.*, 1981; Weiss *et al.*, 1982). An increased risk associated with the therapy was found among women who reported hysterectomy, but the finding was based on very few cases and may have been due to chance. We did not find an increased risk associated with oestrogen therapy for any particular tumour types as suggested by Cramer *et al.* (1981) and Weiss *et al.* (1982).

The evidence linking talc with ovarian cancer is controversial (Anonymous, 1977; Roe, 1979; Longo & Young 1979; Cramer *et al.*, 1982; Hartge *et al.*, 1983). In this study, women who reported talc use in the genital area more than once a week or daily had higher risks of ovarian cancer than women who used talc less frequently. The women were not asked how long they had been using talc. It is possible that because of their symptoms or disease-related pelvic examinations, the frequency of current talc use by the cases may not have reflected their frequency of past use. Since these and other results (Cramer *et al.*, 1982; Hartge *et al.*, 1983) are insufficient to reject an association, further work is needed on the relation between genital use of talc and ovarian cancer.

We would like to thank the Imperial Cancer Research Fund for financing the study, Professor Sir Richard Doll for helpful advice, Professor Christopher Hudson and Dr Marigold Curling for reviewing the histology, Dr Eve Wiltshaw, the consultants, nurses and other staff of the participating hospitals for their co-operation and support of the study, Ann Bateman, Kate Rodriques, Gillian Saunders and Rosalie Thomson for their skilful interviewing, and Nina Saroi for typing the manuscript. Margaret Booth is funded by a grant from the Medical Research Council.

### References

ANNEGERS, J.F., STROM, H., DECKER, D.G., DOCKERTY, M.B. & O'FALLON, W.M. (1979). Ovarian cancer: incidence and case control study. *Cancer*, **43**, 723.

ANONYMOUS (1977). Cosmetic talc powder. Editorial. *Lancet*, **i**, 1348.

BAKER, R.J. & NELDER, J.A. (1978). *The GLIM System Release 3*. Royal Statistical Society: Oxford.

BEAVIS, E.L.G., BROWN, J.B. & SMITH, M.A. (1969). Ovarian function after hysterectomy with conservation of the ovaries in premenopausal women. *J. Obstet. Gynaecol. Br. Cwlth*, **76**, 969.

BOOTH, M. & BERAL, V. (1985). The epidemiology of ovarian cancer. In *Ovarian Cancer*, Hudson, C.N. (ed), p. 22. Oxford University Press: Oxford.

BRESLOW, N.E. & DAY, N.E. (1980). *Statistical Methods in Cancer Research, Vol. 1. The Analysis of Case–control Studies*. IARC Scientific Publications No. 32. IARC: Lyon.

CANCER AND STEROID HORMONE STUDY OF THE CENTRES FOR DISEASE CONTROL AND THE NATIONAL INSTITUTE OF CHILD HEALTH AND HUMAN DEVELOPMENT (1987). The reduction in risk of ovarian cancer associated with oral-contraceptive use. *N. Engl. J. Med.*, **316**, 650.

CASAGRANDE, J.T., PIKE, M.C., ROSS, R.K., LOUIE, E.W., ROY, S. & HENDERSON, B.E. (1979). 'Incessant ovulation' and ovarian cancer. *Lancet*, **ii**, 170.

CRAMER, D.W., DEVESA, S.S. & WELCH, W.R. (1981). Trends in the incidence of endometrioid and clear cell cancers of the ovary in the United States. *Am. J. Epidemiol.*, **114**, 201.

CRAMER, D.W., WELCH, W.R., SCULLY, R.E. & WOJCIECHOWSKI, C.A. (1982). Ovarian cancer and talc: a case-control study. *Cancer*, **50**, 372.

CRAMER, D.W., HUTCHISON, G.B., WELCH, W.R., SCULLY, R.E. & RYAN, K.J. (1983). Determinants of ovarian cancer risk. I. Reproductive experiences and family history. *J. Natl Cancer Inst.*, **71**, 711.

ELLSWORTH, L.R., ALLEN, H.H. & NISKER, J.A. (1983). Ovarian function after radical hysterectomy for Stage 1B carcinoma of cervix. *Am. J. Obstet. Gynecol.*, **145**, 185.

FATHALLA, M.F. (1971). Incessant ovulation–a factor in ovarian neoplasia? *Lancet*, **ii**, 163.

FRANCESCHI, S., LA VECCHIA, C., HELMRICH, S.P., MANGIONI, C. & TOGNONI, G. (1982). Risk factors for epithelial ovarian cancer in Italy. *Am. J. Epidemiol.*, **115**, 714.

HARTGE, P., HOOVER, R., LESHER, L.P. & McGOWAN, L. (1983). Talc and ovarian cancer. *J. Am. Med. Assoc.*, **250**, 1844.

HILDRETH, N.G., KELSEY, J.L., LIVOLSI, V.A. & 5 others (1981). An epidemiologic study of epithelial carcinoma of the ovary. *Am. J. Epidemiol.*, **114**, 398.

JOLY, D.J., LILIENFELD, A.M., DIAMOND, E.L. & BROSS, I.D.J. (1974). An epidemiologic study of the relationship of reproductive experience to cancer of the ovary. *Am. J. Epidemiol.*, **99**, 190.

LESHER, L., McGOWAN, L., HARTGE, P. & HOOVER, R. (1985). Age at first birth and risk of epithelial ovarian cancer. *J. Natl Cancer Inst.*, **74**, 1361.

LONGO, D.L. & YOUNG, R.C. (1979). Cosmetic talc and ovarian cancer. *Lancet*, **ii**, 349.

McGOWAN, L., PARENT, L., LEDNAR, W & NORRIS, H.J. (1979). The woman at risk for developing ovarian cancer. *Gynecol. Oncol.*, **7**, 325.

MORI, M., KIYOSAWA, H. & MIYAKE, H. (1984). Case-control study of ovarian cancer in Japan. *Cancer*, **53**, 2746.

NASCA, P.C., GREENWALD, P., CHOROST, S., RICHART, R., & CAPUTO, T. (1984). An epidemiologic case-control study of ovarian cancer and reproductive factors. *Am. J. Epidemiol.*, **119**, 705.

NEIL, J.R., HAMMOND, G.T., NOBLE, A.D., RUSHTON, L. & LETCHWORTH, A.T. (1975). Late complications of sterilisation by laparoscopy and tubal ligation. *Lancet*, **ii**, 699.

NEWHOUSE, M.L., PEARSON, R.M., FULLERTON, J.M., BOESON, E.A.M. & SHANNON, H.S. (1977). A case control study of carcinoma of the ovary. *Br. J. Prev. Social Med.*, **31**, 148.

OFFICE OF POPULATION CENSUSES AND SURVEYS. (1970). *Classifications of Occupations*. HMSO: London.

RISCH, H.A., WEISS, N.S., LYON, J.L., DALING, J.R., & LIFF, J.M. (1983). Events of reproductive life and the incidence of epithelial ovarian cancer. *Am. J. Epidemiol.*, **117**, 128.

ROE, F.J.C. (1979). Controversy: cosmetic talc and ovarian cancer. *Lancet*, **ii**, 744.

TZONOU, A., DAY, N.E., TRICHOPOULOS, D. & 4 others (1984). The epidemiology of ovarian cancer in Greece: a case-control study. *Eur. J. Cancer Clin. Oncol.*, **20**, 1045.

WEISS, N.S., LYON, J.L., KRISHNAMURTHY, S., DIETERT, S.E., LIFF, J.M. & DALING, J.R. (1982). Noncontraceptive estrogen use and the occurance of ovarian cancer. *J. Natl Cancer Inst.*, **68**, 95.

WEST, R.O. (1966). Epidemiologic study of malignancies of the ovaries. *Cancer*, **19**, 1001.

WU, N.L., WHITTEMORE, A.S., PAFFENBARGER, R.S. & 7 others (1988). Personal and environmental characteristics related to epithelial ovarian cancer. 1. Reproductive and menstrual events and oral contraceptive use. *Am. J. Epidemiol.*, **128**, 1216.

WYNDER, E.L., DODO, H. & BARBER, H.R.K. (1969). Epidemiology of cancer of the ovary. *Cancer*, **23**, 352.